# PD12 Cost Effectiveness Analysis Of Nivolumab Plus Ipilimumab For First-Line Treatment Of Advanced Non-Small Cell Lung Cancer In China

**DOI:** 10.1017/S0266462324002800

**Published:** 2025-01-07

**Authors:** Dunming Xiao, Yingyao Chen, Yi Yang

## Abstract

**Introduction:**

Non-small cell lung cancer (NSCLC) is the most prevalent malignant tumor in China. This study aimed to compare the cost effectiveness of combined nivolumab and ipilimumab with chemotherapy as a first-line treatment for advanced NSCLC. The findings will contribute to the economic evidence for making clinical and health policy decisions.

**Methods:**

Taking a healthcare system perspective, this study used a partitioned survival analysis model to simulate the disease trajectory of advanced NSCLC during first-line treatment over a model cycle of three weeks. The simulation extended over a span of 12 years. A five percent discount was incorporated for both costs and health outcomes. Published clinical efficacy and cost data were extracted from the CheckMate 9LA study (NCT03215706) and drug pricing information was gathered from the YaoZhi website. Utility values were derived from 13 tertiary hospitals in five provinces of China. Base case and sensitivity analyses were also conducted.

**Results:**

The combination of nivolumab and ipilimumab resulted in a lifetime cost of CNY850,068 (USD119,127) and 1.796 quality-adjusted life-years (QALYs), whereas chemotherapy incurred a lifetime cost of CNY276,313 (USD38,722) and a gain of 1.206 QALYs. The incremental cost-effectiveness ratio (ICER) for combination therapy was CNY971,955 (USD136,208) per QALY gained, which was more than three times the average gross domestic product per capita in China (CNY85,698 [USD12,010] in 2022) and indicated that the therapy was not cost effective. Probabilistic sensitivity analysis indicated that the likelihood of nivolumab plus ipilimumab being cost effective, compared with chemotherapy, was 0.02.

**Conclusions:**

Nivolumab plus ipilimumab demonstrated enhanced health outcomes for patients with advanced NSCLC, compared with standard chemotherapy, but the ICER exceeded the acceptable threshold, suggesting that the treatment is not cost effective.

